# Crystal structure of 2-(2,3-di­meth­oxy­naphthalen-1-yl)-3-hy­droxy-6-meth­oxy-4*H*-chromen-4-one

**DOI:** 10.1107/S2056989015018861

**Published:** 2015-10-14

**Authors:** Seunghyun Ahn, Yoongho Lim, Dongsoo Koh

**Affiliations:** a Division of Bioscience and Biotechnology, BMIC, Konkuk University, Seoul 143-701, Republic of Korea; b Department of Applied Chemistry, Dongduk Women’s University, Seoul 136-714, Republic of Korea

**Keywords:** crystal structure, flavonol, hydrogen bonding, fluorescence

## Abstract

In the title compound, C_22_H_18_O_6_, the dimeth­oxy-substituted naphthalene ring system is twisted relative to the 4*H*-chromenon skeleton by 88.96 (3)°. The two meth­oxy substituents are tilted from the naphthalene ring system by 1.4 (4) and 113.0 (2)°, respectively. An intra­molecular O—H⋯O hydrogen bond closes an *S*(5) ring motif. In the crystal, pairs of O—H⋯O hydrogen bonds form inversion dimers with *R* ^2^_2_(10) loops and C—H⋯O inter­actions connect the dimers into [010] chains.

## Related literature   

For the synthesis and biological properties of flavonols, see: Burmistrova *et al.* (2014 ▸); Lee *et al.* (2014 ▸); Dias *et al.* (2013 ▸); Yong *et al.* (2013 ▸); Klymchenko *et al.* (2003 ▸). For flavonols in natural products, see: Bendaikha *et al.* (2014 ▸); Prescott *et al.* (2013 ▸). For related structures, see: Narita *et al.* (2015 ▸); Yoo *et al.* (2014 ▸); Serdiuk *et al.* (2013 ▸).
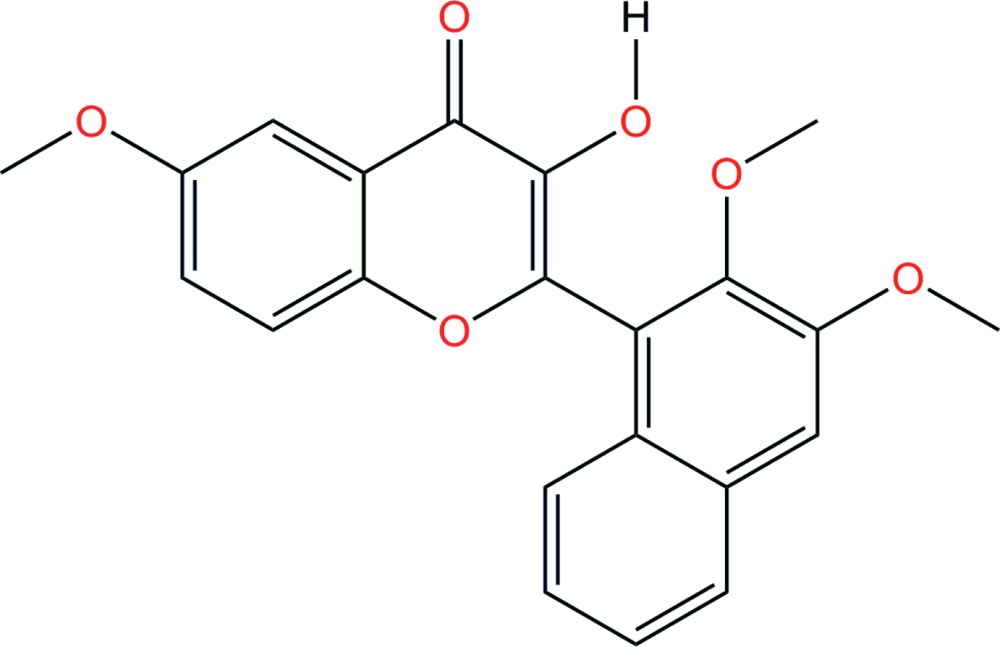


## Experimental   

### Crystal data  

C_22_H_18_O_6_
*M* *_r_* = 378.36
Monoclinic, 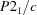

*a* = 11.8571 (12) Å
*b* = 9.0888 (9) Å
*c* = 17.3977 (17) Å
β = 95.253 (2)°
*V* = 1867.0 (3) Å^3^
*Z* = 4
Mo *K*α radiation
μ = 0.10 mm^−1^
*T* = 200 K
0.19 × 0.11 × 0.05 mm

### Data collection  

Bruker SMART CCD area-detector diffractometer
13406 measured reflections
4625 independent reflections
2786 reflections with *I* > 2σ(*I*)
*R*_int_ = 0.036

### Refinement  

*R*[*F* ^2^ > 2σ(*F* ^2^)] = 0.055
*wR*(*F* ^2^) = 0.184
*S* = 1.11
4625 reflections
257 parameters
H-atom parameters constrained
Δρ_max_ = 0.28 e Å^−3^
Δρ_min_ = −0.30 e Å^−3^

### 

Data collection: *SMART* (Bruker, 2000 ▸); cell refinement: *SAINT* (Bruker, 2000 ▸); data reduction: *SAINT*; program(s) used to solve structure: *SHELXS97* (Sheldrick, 2008 ▸); program(s) used to refine structure: *SHELXL97* (Sheldrick, 2008 ▸); molecular graphics: *SHELXTL* (Sheldrick, 2008 ▸); software used to prepare material for publication: *SHELXTL*.

## Supplementary Material

Crystal structure: contains datablock(s) I. DOI: 10.1107/S2056989015018861/ff2142sup1.cif

Structure factors: contains datablock(s) I. DOI: 10.1107/S2056989015018861/ff2142Isup2.hkl

Supporting information file. DOI: 10.1107/S2056989015018861/ff2142Isup3.cml

**Fig. 1.**
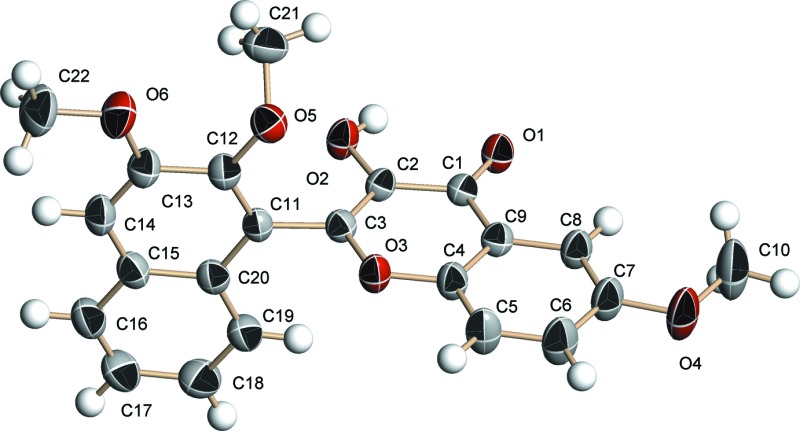
Molecular structure of the title compound, showing the atom-labelling scheme and with displacement ellipsoids drawn at the 50% probability level.

. DOI: 10.1107/S2056989015018861/ff2142fig1.tif
Mol­ecular structure of the title compound, showing the atom-labelling scheme and with displacement ellipsoids drawn at the 50% probability level.

**Fig. 2.**
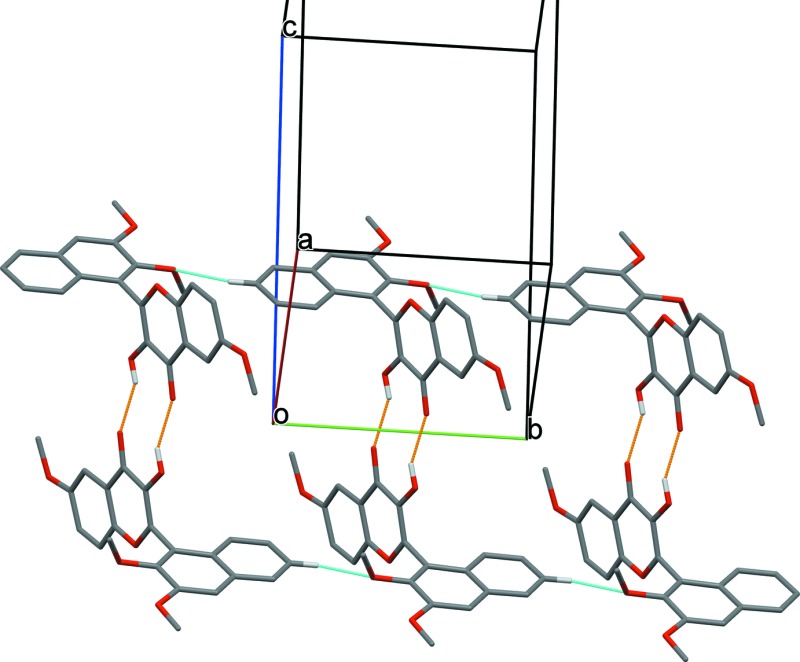
Part of the crystal structure with intermolecular O—H···O hydrogen bonds shown as brown dashed lines and C—H···O interactions shown as blue dashed lines.

. DOI: 10.1107/S2056989015018861/ff2142fig2.tif
Part of the crystal structure with inter­molecular O—H⋯O hydrogen bonds shown as brown dashed lines and C—H⋯O inter­actions shown as blue dashed lines.

**Fig. 3.**
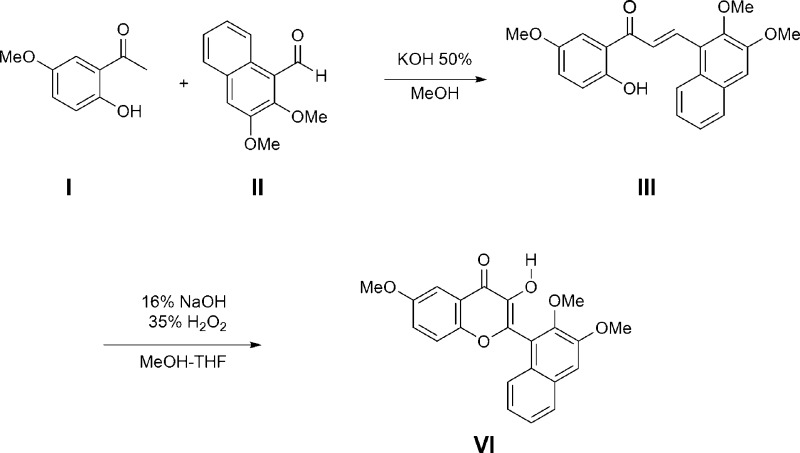
Synthetic scheme for the title compound.

. DOI: 10.1107/S2056989015018861/ff2142fig3.tif
Synthetic scheme for the title compound.

CCDC reference: 1430031

Additional supporting information:  crystallographic information; 3D view; checkCIF report

## Figures and Tables

**Table 1:** Hydrogen-bond geometry (, )

*D*H*A*	*D*H	H*A*	*D* *A*	*D*H*A*
O2H2O1	0.84	2.32	2.750(2)	112
O2H2O1^i^	0.84	2.02	2.761(2)	146
C14H14O1^ii^	0.95	2.60	3.502(3)	158
C17H17O5^iii^	0.95	2.60	3.342(3)	136
C22H22*A*O1^iv^	0.98	2.58	3.509(4)	159

Symmetry codes: (i) 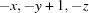; (ii) 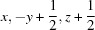; (iii) 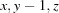; (iv) 
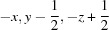
.

## supplementary crystallographic information

### S1. Introduction

Flavonols, such as Quercetin, Aza­leatin and Kaempferol, are a class of flavonoids that have a 3-hy­droxy­flavone backbone. Because of their wide spectrum of biological activities (Burmistrova *et al.* 2014, Dias *et al.* 2013), variety of flanonols have been isolated from natural sources and synthesized (Bendaikha *et al.* 2014; Prescott *et al.* 2013). In addition, they have been used as fluorescent probes for sensing and imaging due to their dual fluorescence. The fluorescence of flavonols has been shown to be related to the angle between the 4*H*-chromene-4-one moiety and the attached aromatic ring (Klymchenko *et al.* 2003). Our research project has been focused on development of novel flavonols which show broad range of biological activities (Lee *et al.* 2014), therefore the title compound was synthesized and its crystal structure was determined. A starting material, chalcone (III), was prepared by the previously reported methods (Yong *et al.* 2013). Flavonol was obtained by oxidative cyclization of the chalcone (III) with H_2_O_2_ in alkaline methanol medium (Fig. 3). In the title compound, C_22_H_18_O_6_, angle between the di­meth­oxy-substituted naphthalene ring and the 4*H*-chromenon skeleton is 88.96 (3)°, which shows they are almost orthogonal each other. In our previous report on flavonol (Yoo *et al.*, 2014), the angle between 4*H*-chromenon and benzene ring is 5.2 (4)°. The meth­oxy groups in naphthalene ring at C12 and C13 are tilted from naphthalene ring by 1.4 (4)° and 113.0 (2)°, respectively. Meth­oxy group at C12 (*meta* position) lies almost in the same plane of naphthalene ring. Meth­oxy group at C13 (*ortho* position), however, is twisted away from the plane of naphthalene ring. An intra­molecular O—H···O hydrogen bond closes S(5) ring motif. In the crystal, pairs of O—H···O hydrogen bonds form inversion dimer with graph-set notation *R*^2^_2_(10) and C—H···O inter­actions connect the dimers into [010] chains. Examples of structures of flavonols have been published (Narita *et al.*, 2015; Serdiuk *et al.*, 2013).

### S2. Experimental

Equivalent amount of 2-hy­droxy-5-meth­oxy­aceto­phenone (I, 10 mmol, 1.66 g) and 2,3-di-meth­oxy­naphthaldehyde (II, 10 mmol, 2.16 g) were dissolved in 20 ml of methanol and the temperature was adjusted to around 2–4 °C in an ice-bath. To a cooled reaction mixture was added 2 ml of 50% (*w*/*v*) aq. KOH solution and stirred at room temperature for 20 h. At the end of the reaction, ice-water was added to the mixture and acidified with 3 N HCl (pH = 3–4). The precipitation was filtered under vacuum and washed with methanol to give chalcone compound III (yield: 48%, m.p: 407–408 K). The chalcone compound (III, 1 mmol, 364 mg) was dissolved in 6 ml of methanol and 4 ml of THF. The reaction was cooled in a water-ice bath (2–4 °C) and a cold solution of 16% sodium hydroxide (1 ml) was added with stirring. After 10 min, to the reaction mixture was added 2 ml of 35% H_2_O_2_. The end point of reaction was monitored by TLC. After completion of reaction, the reaction mixture was acidified with 3 N HCl (pH = 4–5). The pale yellow precipitate obtained was filtered and washed with ethanol to give the titled compound (66%). Recrystallization in the ethanol solvent gave crystals (mp: 573–574 K)

### S2.1. Refinement

The H atoms were placed at calculated positions and refined as riding with C–H = 0.95 A [*U*_iso_(H) = 1.2 *U*_eq_(C)].

### Crystal data

**Table:**

C_22_H_18_O_6_	*F*(000) = 792
*M**_r_* = 378.36	*D*_x_ = 1.346 Mg m^−^^3^
Monoclinic, *P*2_1_/*c*	Mo *K*α radiation, λ = 0.71073 Å
Hall symbol: -P 2ybc	Cell parameters from 4844 reflections
*a* = 11.8571 (12) Å	θ = 2.4–28.2°
*b* = 9.0888 (9) Å	µ = 0.10 mm^−^^1^
*c* = 17.3977 (17) Å	*T* = 200 K
β = 95.253 (2)°	Block, yellow
*V* = 1867.0 (3) Å^3^	0.19 × 0.11 × 0.05 mm
*Z* = 4	

### Data collection

**Table:**

Bruker SMART CCD area-detector diffractometer	2786 reflections with *I* > 2σ(*I*)
Radiation source: fine-focus sealed tube	*R*_int_ = 0.036
Graphite monochromator	θ_max_ = 28.3°, θ_min_ = 1.7°
phi and ω scans	*h* = −15→10
13406 measured reflections	*k* = −11→12
4625 independent reflections	*l* = −22→23

### Refinement

**Table:**

Refinement on *F*^2^	Primary atom site location: structure-invariant direct methods
Least-squares matrix: full	Secondary atom site location: difference Fourier map
*R*[*F*^2^ > 2σ(*F*^2^)] = 0.055	Hydrogen site location: inferred from neighbouring sites
*wR*(*F*^2^) = 0.184	H-atom parameters constrained
*S* = 1.11	*w* = 1/[σ^2^(*F*_o_^2^) + (0.0751*P*)^2^ + 0.4684*P*] where *P* = (*F*_o_^2^ + 2*F*_c_^2^)/3
4625 reflections	(Δ/σ)_max_ < 0.001
257 parameters	Δρ_max_ = 0.28 e Å^−^^3^
0 restraints	Δρ_min_ = −0.29 e Å^−^^3^

### Special details

**Table:**

Geometry. All e.s.d.'s (except the e.s.d. in the dihedral angle between two l.s. planes) are estimated using the full covariance matrix. The cell e.s.d.'s are taken into account individually in the estimation of e.s.d.'s in distances, angles and torsion angles; correlations between e.s.d.'s in cell parameters are only used when they are defined by crystal symmetry. An approximate (isotropic) treatment of cell e.s.d.'s is used for estimating e.s.d.'s involving l.s. planes.
Refinement. Refinement of *F*^2^ against ALL reflections. The weighted *R*-factor *wR* and goodness of fit *S* are based on *F*^2^, conventional *R*-factors *R* are based on *F*, with *F* set to zero for negative *F*^2^. The threshold expression of *F*^2^ > σ(*F*^2^) is used only for calculating *R*-factors(gt) *etc*. and is not relevant to the choice of reflections for refinement. *R*-factors based on *F*^2^ are statistically about twice as large as those based on *F*, and *R*- factors based on ALL data will be even larger.

### Fractional atomic coordinates and isotropic or equivalent isotropic displacement parameters (Å^2^)

**Table:**

	*x*	*y*	*z*	*U*_iso_*/*U*_eq_	
C1	0.19294 (16)	0.5697 (2)	0.04954 (11)	0.0324 (4)	
O1	0.12049 (12)	0.59265 (17)	−0.00540 (8)	0.0419 (4)	
C2	0.16533 (16)	0.4835 (2)	0.11528 (11)	0.0334 (4)	
O2	0.05942 (12)	0.42964 (18)	0.11735 (9)	0.0444 (4)	
H2	0.0202	0.4519	0.0763	0.067*	
C3	0.24396 (16)	0.4529 (2)	0.17434 (12)	0.0346 (5)	
O3	0.35228 (12)	0.50146 (17)	0.17607 (8)	0.0430 (4)	
C4	0.38419 (18)	0.5844 (2)	0.11559 (13)	0.0425 (5)	
C5	0.4958 (2)	0.6288 (3)	0.11996 (15)	0.0592 (7)	
H5	0.5467	0.6023	0.1631	0.071*	
C6	0.5324 (2)	0.7113 (3)	0.06168 (16)	0.0659 (8)	
H6	0.6096	0.7402	0.0638	0.079*	
C7	0.45694 (19)	0.7541 (3)	−0.00160 (15)	0.0533 (7)	
C8	0.34661 (18)	0.7105 (3)	−0.00567 (13)	0.0434 (5)	
H8	0.2958	0.7393	−0.0484	0.052*	
C9	0.30769 (17)	0.6229 (2)	0.05332 (12)	0.0358 (5)	
O4	0.50460 (14)	0.8384 (3)	−0.05520 (11)	0.0737 (6)	
C10	0.4292 (3)	0.8899 (4)	−0.11779 (19)	0.0901 (12)	
H10A	0.3953	0.8057	−0.1465	0.135*	
H10B	0.4711	0.9502	−0.1522	0.135*	
H10C	0.3694	0.9491	−0.0978	0.135*	
C11	0.22233 (16)	0.3626 (2)	0.24206 (12)	0.0353 (5)	
C12	0.18157 (18)	0.4294 (2)	0.30418 (12)	0.0395 (5)	
C13	0.15722 (19)	0.3451 (3)	0.36980 (12)	0.0437 (5)	
C14	0.16900 (19)	0.1962 (3)	0.36856 (13)	0.0457 (6)	
H14	0.1494	0.1399	0.4114	0.055*	
C15	0.20979 (18)	0.1235 (3)	0.30471 (13)	0.0429 (5)	
C16	0.2199 (2)	−0.0312 (3)	0.30177 (16)	0.0541 (6)	
H16	0.1991	−0.0887	0.3438	0.065*	
C17	0.2589 (2)	−0.0992 (3)	0.23987 (17)	0.0620 (7)	
H17	0.2643	−0.2035	0.2388	0.074*	
C18	0.2910 (2)	−0.0160 (3)	0.17768 (17)	0.0600 (7)	
H18	0.3193	−0.0641	0.1349	0.072*	
C19	0.2818 (2)	0.1338 (3)	0.17818 (14)	0.0480 (6)	
H19	0.3042	0.1891	0.1358	0.058*	
C20	0.23973 (17)	0.2073 (2)	0.24075 (12)	0.0394 (5)	
O5	0.16815 (13)	0.57945 (17)	0.30440 (9)	0.0469 (4)	
C21	0.0524 (2)	0.6294 (3)	0.30143 (18)	0.0666 (8)	
H21A	0.0014	0.5443	0.2972	0.100*	
H21B	0.0361	0.6935	0.2565	0.100*	
H21C	0.0411	0.6842	0.3486	0.100*	
O6	0.12322 (16)	0.4267 (2)	0.42964 (9)	0.0579 (5)	
C22	0.1006 (3)	0.3472 (4)	0.49711 (16)	0.0753 (9)	
H22A	0.0346	0.2835	0.4853	0.113*	
H22B	0.0851	0.4166	0.5380	0.113*	
H22C	0.1665	0.2869	0.5145	0.113*	

### Atomic displacement parameters (Å^2^)

**Table:**

	*U*^11^	*U*^22^	*U*^33^	*U*^12^	*U*^13^	*U*^23^
C1	0.0330 (10)	0.0339 (10)	0.0302 (10)	−0.0019 (8)	0.0018 (8)	−0.0013 (8)
O1	0.0368 (8)	0.0538 (9)	0.0339 (8)	−0.0092 (7)	−0.0029 (6)	0.0068 (7)
C2	0.0314 (10)	0.0361 (11)	0.0329 (10)	−0.0056 (8)	0.0047 (8)	0.0003 (8)
O2	0.0315 (8)	0.0612 (10)	0.0395 (8)	−0.0139 (7)	−0.0020 (6)	0.0109 (7)
C3	0.0322 (10)	0.0345 (10)	0.0369 (10)	−0.0035 (8)	0.0018 (8)	0.0025 (8)
O3	0.0312 (8)	0.0541 (10)	0.0426 (8)	−0.0087 (6)	−0.0025 (6)	0.0135 (7)
C4	0.0367 (12)	0.0464 (13)	0.0439 (12)	−0.0070 (9)	0.0018 (9)	0.0111 (10)
C5	0.0349 (12)	0.0815 (19)	0.0585 (15)	−0.0148 (12)	−0.0095 (11)	0.0268 (14)
C6	0.0351 (13)	0.091 (2)	0.0703 (18)	−0.0192 (13)	−0.0024 (12)	0.0313 (16)
C7	0.0389 (13)	0.0680 (17)	0.0532 (14)	−0.0144 (11)	0.0047 (10)	0.0190 (12)
C8	0.0355 (12)	0.0540 (14)	0.0404 (12)	−0.0096 (10)	0.0017 (9)	0.0095 (10)
C9	0.0333 (11)	0.0369 (11)	0.0371 (11)	−0.0054 (8)	0.0024 (8)	0.0000 (9)
O4	0.0433 (10)	0.1098 (17)	0.0676 (12)	−0.0240 (10)	0.0024 (8)	0.0421 (12)
C10	0.0637 (19)	0.128 (3)	0.076 (2)	−0.0287 (19)	−0.0054 (16)	0.059 (2)
C11	0.0290 (10)	0.0396 (11)	0.0366 (11)	−0.0044 (8)	−0.0010 (8)	0.0072 (9)
C12	0.0365 (11)	0.0424 (12)	0.0388 (11)	−0.0050 (9)	−0.0014 (9)	0.0044 (9)
C13	0.0448 (13)	0.0507 (14)	0.0353 (11)	−0.0063 (10)	0.0018 (9)	0.0038 (10)
C14	0.0436 (13)	0.0549 (14)	0.0378 (12)	−0.0068 (10)	0.0001 (9)	0.0126 (10)
C15	0.0370 (12)	0.0431 (12)	0.0476 (13)	−0.0033 (9)	−0.0026 (9)	0.0113 (10)
C16	0.0561 (15)	0.0451 (14)	0.0611 (16)	−0.0010 (11)	0.0048 (12)	0.0147 (12)
C17	0.0683 (18)	0.0414 (14)	0.0770 (19)	0.0033 (12)	0.0113 (15)	0.0110 (13)
C18	0.0646 (17)	0.0470 (15)	0.0698 (18)	0.0048 (12)	0.0144 (13)	−0.0025 (13)
C19	0.0478 (13)	0.0444 (13)	0.0521 (14)	0.0010 (10)	0.0065 (10)	0.0069 (11)
C20	0.0333 (11)	0.0415 (12)	0.0425 (12)	−0.0022 (9)	−0.0014 (9)	0.0061 (10)
O5	0.0501 (10)	0.0394 (9)	0.0511 (9)	−0.0035 (7)	0.0040 (7)	0.0007 (7)
C21	0.0572 (17)	0.0562 (16)	0.088 (2)	0.0128 (13)	0.0158 (14)	0.0053 (15)
O6	0.0759 (12)	0.0630 (12)	0.0363 (9)	−0.0054 (9)	0.0126 (8)	0.0012 (8)
C22	0.099 (2)	0.087 (2)	0.0412 (14)	0.0023 (18)	0.0173 (14)	0.0098 (15)

### Geometric parameters (Å, º)

**Table:**

C1—O1	1.243 (2)	C12—O5	1.373 (3)
C1—C9	1.440 (3)	C12—C13	1.426 (3)
C1—C2	1.448 (3)	C13—C14	1.361 (3)
C2—O2	1.351 (2)	C13—O6	1.369 (3)
C2—C3	1.352 (3)	C14—C15	1.415 (3)
O2—H2	0.8400	C14—H14	0.9500
C3—O3	1.356 (2)	C15—C16	1.413 (3)
C3—C11	1.477 (3)	C15—C20	1.420 (3)
O3—C4	1.375 (2)	C16—C17	1.359 (4)
C4—C5	1.379 (3)	C16—H16	0.9500
C4—C9	1.392 (3)	C17—C18	1.401 (4)
C5—C6	1.363 (3)	C17—H17	0.9500
C5—H5	0.9500	C18—C19	1.366 (3)
C6—C7	1.409 (3)	C18—H18	0.9500
C6—H6	0.9500	C19—C20	1.407 (3)
C7—C8	1.362 (3)	C19—H19	0.9500
C7—O4	1.368 (3)	O5—C21	1.442 (3)
C8—C9	1.410 (3)	C21—H21A	0.9800
C8—H8	0.9500	C21—H21B	0.9800
O4—C10	1.423 (3)	C21—H21C	0.9800
C10—H10A	0.9800	O6—C22	1.425 (3)
C10—H10B	0.9800	C22—H22A	0.9800
C10—H10C	0.9800	C22—H22B	0.9800
C11—C12	1.366 (3)	C22—H22C	0.9800
C11—C20	1.427 (3)		
			
O1—C1—C9	124.23 (18)	C11—C12—C13	120.4 (2)
O1—C1—C2	120.52 (18)	O5—C12—C13	120.0 (2)
C9—C1—C2	115.25 (16)	C14—C13—O6	126.1 (2)
O2—C2—C3	118.88 (18)	C14—C13—C12	119.5 (2)
O2—C2—C1	119.66 (16)	O6—C13—C12	114.4 (2)
C3—C2—C1	121.45 (18)	C13—C14—C15	121.3 (2)
C2—O2—H2	109.5	C13—C14—H14	119.3
C2—C3—O3	122.40 (18)	C15—C14—H14	119.3
C2—C3—C11	124.24 (18)	C16—C15—C14	122.0 (2)
O3—C3—C11	113.35 (16)	C16—C15—C20	118.5 (2)
C3—O3—C4	119.20 (15)	C14—C15—C20	119.5 (2)
O3—C4—C5	116.63 (19)	C17—C16—C15	121.2 (2)
O3—C4—C9	121.86 (18)	C17—C16—H16	119.4
C5—C4—C9	121.5 (2)	C15—C16—H16	119.4
C6—C5—C4	119.3 (2)	C16—C17—C18	120.2 (2)
C6—C5—H5	120.4	C16—C17—H17	119.9
C4—C5—H5	120.4	C18—C17—H17	119.9
C5—C6—C7	120.7 (2)	C19—C18—C17	120.4 (3)
C5—C6—H6	119.7	C19—C18—H18	119.8
C7—C6—H6	119.7	C17—C18—H18	119.8
C8—C7—O4	125.6 (2)	C18—C19—C20	120.8 (2)
C8—C7—C6	119.9 (2)	C18—C19—H19	119.6
O4—C7—C6	114.5 (2)	C20—C19—H19	119.6
C7—C8—C9	120.2 (2)	C19—C20—C15	118.9 (2)
C7—C8—H8	119.9	C19—C20—C11	122.9 (2)
C9—C8—H8	119.9	C15—C20—C11	118.1 (2)
C4—C9—C8	118.37 (18)	C12—O5—C21	115.00 (18)
C4—C9—C1	119.78 (19)	O5—C21—H21A	109.5
C8—C9—C1	121.83 (18)	O5—C21—H21B	109.5
C7—O4—C10	115.85 (19)	H21A—C21—H21B	109.5
O4—C10—H10A	109.5	O5—C21—H21C	109.5
O4—C10—H10B	109.5	H21A—C21—H21C	109.5
H10A—C10—H10B	109.5	H21B—C21—H21C	109.5
O4—C10—H10C	109.5	C13—O6—C22	116.3 (2)
H10A—C10—H10C	109.5	O6—C22—H22A	109.5
H10B—C10—H10C	109.5	O6—C22—H22B	109.5
C12—C11—C20	120.96 (19)	H22A—C22—H22B	109.5
C12—C11—C3	118.94 (19)	O6—C22—H22C	109.5
C20—C11—C3	120.05 (19)	H22A—C22—H22C	109.5
C11—C12—O5	119.55 (19)	H22B—C22—H22C	109.5
			
O1—C1—C2—O2	−1.1 (3)	C2—C3—C11—C20	90.0 (3)
C9—C1—C2—O2	179.49 (18)	O3—C3—C11—C20	−89.0 (2)
O1—C1—C2—C3	177.6 (2)	C20—C11—C12—O5	178.55 (17)
C9—C1—C2—C3	−1.8 (3)	C3—C11—C12—O5	−4.0 (3)
O2—C2—C3—O3	179.25 (18)	C20—C11—C12—C13	1.0 (3)
C1—C2—C3—O3	0.6 (3)	C3—C11—C12—C13	178.53 (18)
O2—C2—C3—C11	0.3 (3)	C11—C12—C13—C14	−3.6 (3)
C1—C2—C3—C11	−178.38 (19)	O5—C12—C13—C14	178.93 (19)
C2—C3—O3—C4	−0.3 (3)	C11—C12—C13—O6	176.38 (19)
C11—C3—O3—C4	178.71 (19)	O5—C12—C13—O6	−1.1 (3)
C3—O3—C4—C5	−179.0 (2)	O6—C13—C14—C15	−177.1 (2)
C3—O3—C4—C9	1.5 (3)	C12—C13—C14—C15	2.8 (3)
O3—C4—C5—C6	180.0 (3)	C13—C14—C15—C16	−178.5 (2)
C9—C4—C5—C6	−0.6 (4)	C13—C14—C15—C20	0.4 (3)
C4—C5—C6—C7	1.5 (5)	C14—C15—C16—C17	179.8 (2)
C5—C6—C7—C8	−1.3 (5)	C20—C15—C16—C17	0.9 (4)
C5—C6—C7—O4	179.1 (3)	C15—C16—C17—C18	0.7 (4)
O4—C7—C8—C9	179.7 (3)	C16—C17—C18—C19	−1.0 (4)
C6—C7—C8—C9	0.1 (4)	C17—C18—C19—C20	−0.2 (4)
O3—C4—C9—C8	178.8 (2)	C18—C19—C20—C15	1.8 (3)
C5—C4—C9—C8	−0.6 (4)	C18—C19—C20—C11	−176.3 (2)
O3—C4—C9—C1	−2.9 (3)	C16—C15—C20—C19	−2.1 (3)
C5—C4—C9—C1	177.7 (2)	C14—C15—C20—C19	179.0 (2)
C7—C8—C9—C4	0.8 (4)	C16—C15—C20—C11	176.02 (19)
C7—C8—C9—C1	−177.4 (2)	C14—C15—C20—C11	−2.8 (3)
O1—C1—C9—C4	−176.5 (2)	C12—C11—C20—C19	−179.8 (2)
C2—C1—C9—C4	2.9 (3)	C3—C11—C20—C19	2.7 (3)
O1—C1—C9—C8	1.7 (3)	C12—C11—C20—C15	2.1 (3)
C2—C1—C9—C8	−178.8 (2)	C3—C11—C20—C15	−175.34 (18)
C8—C7—O4—C10	3.5 (5)	C11—C12—O5—C21	113.0 (2)
C6—C7—O4—C10	−176.8 (3)	C13—C12—O5—C21	−69.5 (3)
C2—C3—C11—C12	−87.5 (3)	C14—C13—O6—C22	1.4 (3)
O3—C3—C11—C12	93.5 (2)	C12—C13—O6—C22	−178.6 (2)

### Hydrogen-bond geometry (Å, º)

**Table:**

*D*—H···*A*	*D*—H	H···*A*	*D*···*A*	*D*—H···*A*
O2—H2···O1	0.84	2.32	2.750 (2)	112
O2—H2···O1^i^	0.84	2.02	2.7613 (19)	146
C14—H14···O1^ii^	0.95	2.60	3.502 (3)	158
C17—H17···O5^iii^	0.95	2.60	3.342 (3)	136
C22—H22*A*···O1^iv^	0.98	2.58	3.509 (4)	159

Symmetry codes: (i) −*x*, −*y*+1, −*z*; (ii) *x*, −*y*+1/2, *z*+1/2; (iii) *x*, *y*−1, *z*; (iv) −*x*, *y*−1/2, −*z*+1/2.
