# Supplementary figures and images for: Crystal structure of 2-(2,3-di­meth­oxy­naphthalen-1-yl)-3-hy­droxy-6-meth­oxy-4H-chromen-4-one
Source: Acta Crystallogr E Crystallogr Commun. 2015 Oct 14;71(Pt 11):o842–3. doi: 10.1107/S2056989015018861 (PMC4645031; doi:10.1107/S2056989015018861)

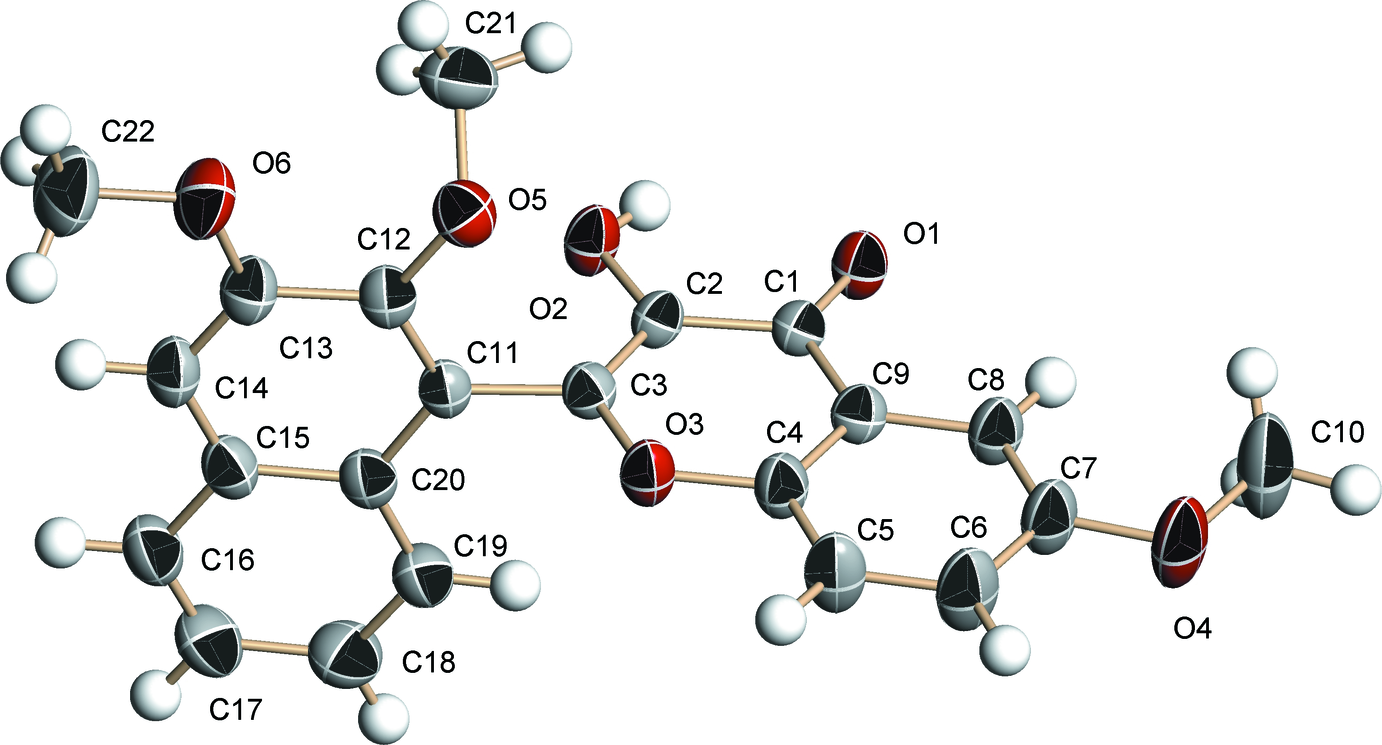

Supplement: Supplementary file 4 [file e-71-0o842-fig1.tif]

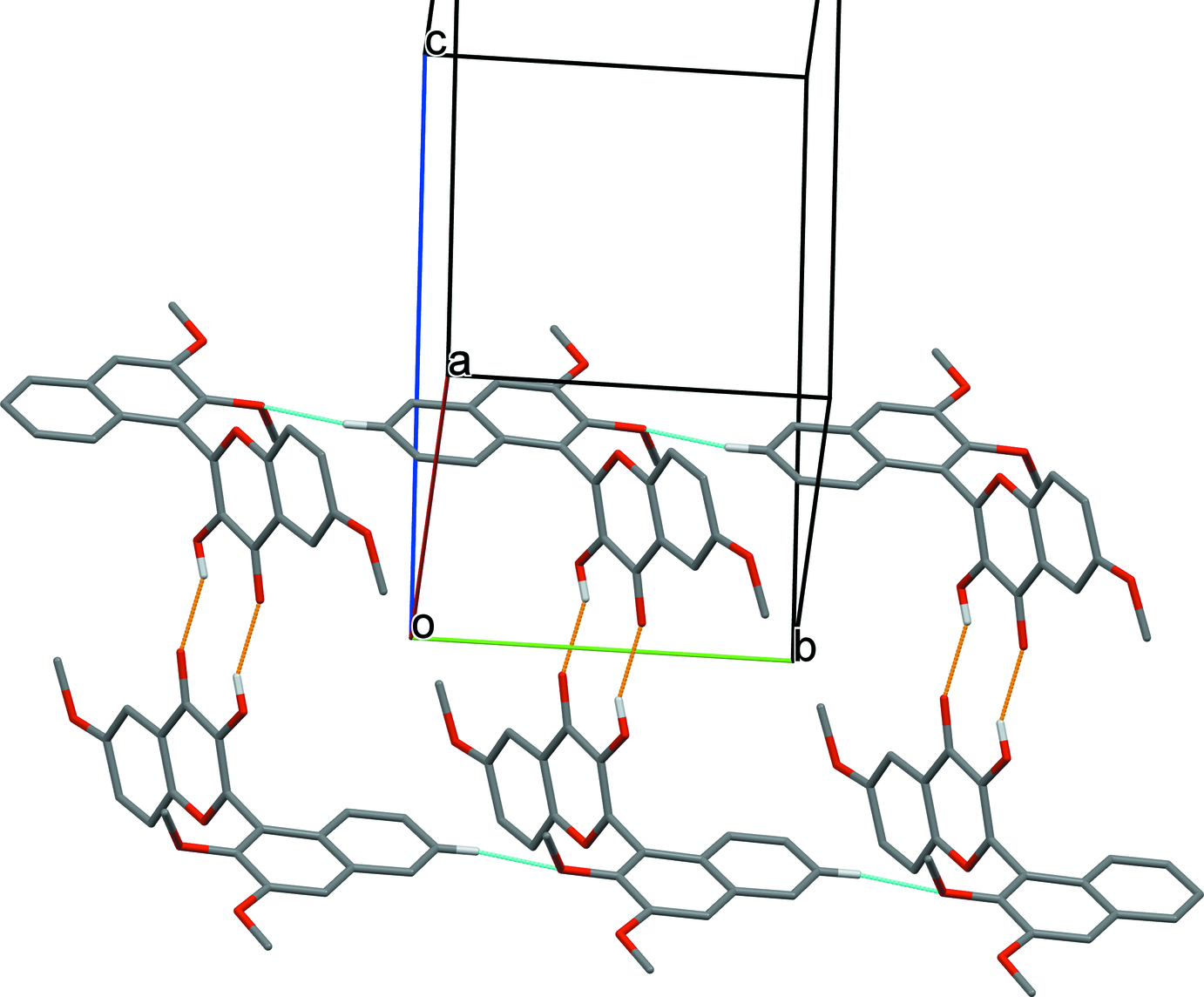

Supplement: Supplementary file 5 [file e-71-0o842-fig2.tif]
